# Supplementary material for: Temperature- versus precipitation-limitation shape local temperature tolerance in a Holarctic freshwater crustacean
Source: Proc Biol Sci. 2019 Jul 24;286(1907):20190929. doi: 10.1098/rspb.2019.0929 (PMC6661336; doi:10.1098/rspb.2019.0929)
Supplement: Temperature treatments across the experimental period for the entire temperature assays. [file rspb20190929supp1.pdf]

Temperature- versus precipitation-limitation shape local temperature tolerance in a Holarctic freshwater crustacean

by Leonie Seefeldt & Dieter Ebert

Proceedings of the Royal Society, B (2019)
DOI: 10.1098/rspb.2019.0929

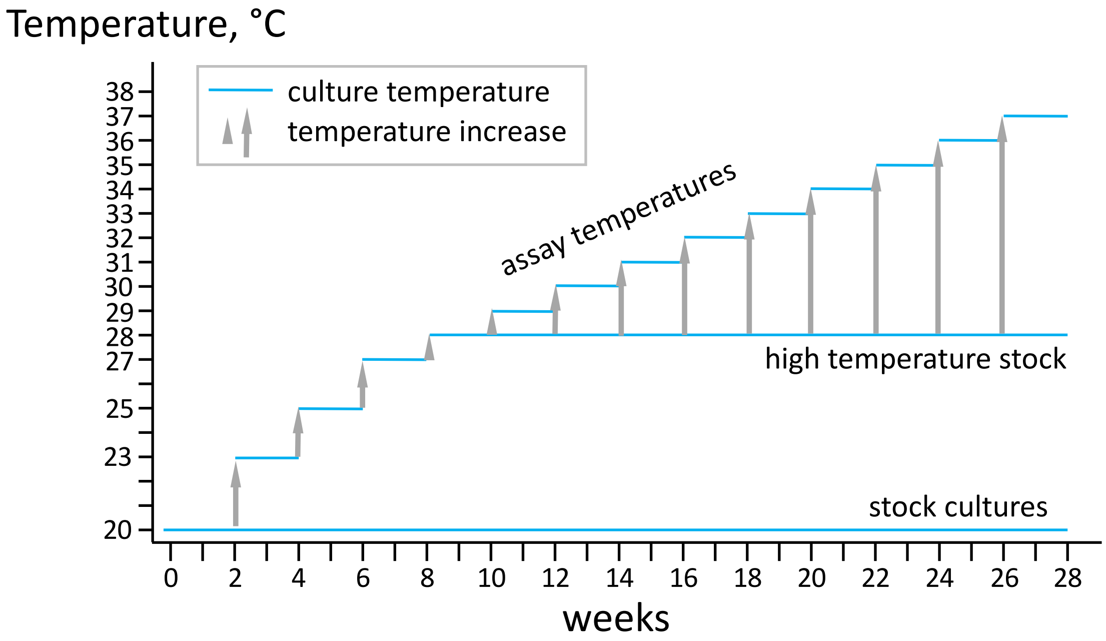

Supplementary figure 1. Temperature treatments across the experimental period. At the beginning, animals were taken from the stock cultures (at 20 °C) and cultured in 380-mL jars filled with 350 mL Daphnia medium at 23 °C. During the acclimation phase, every two weeks, the temperature was raised (23, 25, 26, 27 and 28 °C), and population survival and the presence of juveniles (as an indicator of successful reproduction) were recorded. All replicate populations that were able to reproduce were then kept at 28 °C (high temperature stock). We then took animals from these 28 °C cultures and tested for reproduction and survival at nine higher temperatures (29 to 37 °C). For each of these higher temperatures, we collected three juvenile females (1 – 3 days old) from each of the 5 replicate populations kept at 28 °C and placed them in a 100-mL jar with 80 mL medium. These jars were kept in a walk-in chamber at the higher temperature (assay temperature). Animals were checked for survival and reproduction. If at least one animal of a replicate survived the 14-day assay period, three new juveniles from the same replicates of the high temperature stock were tested at the next higher temperature. If none of the animals survived 14 days at a certain temperature, this replicate was still tested at the next higher temperature. If all animals died again, we assumed that we had exceeded its upper temperature tolerance and discontinued with this replicate. The most heat tolerant animals died at 35 °C, but were still tested at 36 and 37 °C.
